# Holistic management of chronic musculoskeletal pain among elderly patients: A primary care approach

**DOI:** 10.51866/lte.499r

**Published:** 2024-05-21

**Authors:** Trina Sengupta, Riya Halder

**Affiliations:** 1 MBBS, MD, Department of Preventive and Social Medicine, All India Institute of Hygiene and Public Health 110, Chittaranjan Avenue, Kolkata, India. Email: riyahalder94@gmail.com; 2 MBBS, MD, Department of Preventive and Social Medicine, All India Institute of Hygiene and Public Health 110, Chittaranjan Avenue, Kolkata, India.

**Keywords:** Chronic pain, Elderly, India, Primary health care

**Dear editor,**

We are grateful for the response to and interest in our recently published article.^1^ We sincerely endeavour to clarify the queries forwarded to us regarding our article through this letter. Notably, the objectives of our study were to assess the burden of chronic musculoskeletal pain (MSP) among the elderly population and delineate patients’ perspectives towards the treatment and management of MSP using a mixed-method approach.^2^ The qualitative component of the study was conducted to determine how elderly patients experiencing chronic MSP feel and think about the treatment available to them and how they approach their pain management.

It is agreeable that several reasons may cause chronic MSP However, our study did not aim to determine the aetiologies of chronic MSP We comply with the fact that assessing the underlying cause of MSP is imperative to streamline the treatment for each elderly patient. In our study, we focused on a holistic public health approach for managing elderly patients at the primary level of health care as well as for underlining the importance of their overall knowledge about pain medication and the detrimental effects of over-the-counter (OTC) drugs. The usage of OTC drugs may be one of the reasons for aggravating MSP Studies suggest that people using OTC drugs, especially analgesics, may need more effective treatments for chronic pain.^3^ Thus, the overall burden of chronic MSP among elderly patients, irrespective of aetiology, was assessed according to the objective of our study. Among the study participants, 57% reported chronic MSP. The factors that were found to be associated with chronic MSP were increasing age, presence of comorbidity, OTC drug use and depression. These significantly associated factors indicate the worsening nature of chronic MSP among elderly individuals and the lack of awareness regarding the use of OTC drugs.

In conclusion, our study intended to capture the current status of chronic MSP among elderly individuals residing in a rural community in India. A mixed-method approach was applied to assess the overall perspective and awareness of the patients about the management of MSP Similar parameters were also investigated through the quantitative component of the study and explained in the joint display. Therefore, it was beyond the scope of our study to delineate the underlying pathology of chronic MSP. Nonetheless, we agree that for providing tailor-made treatment to every patient with chronic MSP, it is essential to know the aetiology of the disease. However, our findings suggest that strengthening the primary level of care, including preventive and health-promotive approaches, among elderly individuals experiencing chronic MSP should be the foremost priority in resource-poor settings of countries such as India.

## References

[1] Finsterer J (2024). For optimal treatment of musculoskeletal pain among elderly individuals, clarification of its aetiology is essential.. Malays Fam Physician..

[2] Sengupta T, Paul B, Banerjee A, Das R, Halder R (2023). Chronic musculoskeletal pain among elderly individuals in a rural area of West Bengal: a mixed-method study.. Malays Fam Physician..

[3] Abbott FV, Fraser MI (1998). Use and abuse of over-the-counter analgesic agents.. J Psychiatry Neurosci..

